# Hyaluronic Acid—Dexamethasone Nanoparticles for Local Adjunct Therapy of Lung Inflammation

**DOI:** 10.3390/ijms221910480

**Published:** 2021-09-28

**Authors:** Candelaria Ines Camara, Laura Bertocchi, Caterina Ricci, Rosaria Bassi, Annalisa Bianchera, Laura Cantu’, Ruggero Bettini, Elena Del Favero

**Affiliations:** 1 Department of Medical Biotechnology and Translational Medicine, Università degli Studi di Milano, LITA, Via Fratelli Cervi 93, 20090 Segrate, Italy; candelaria_camara@unc.edu.ar (C.I.C.); caterina.ricci@unimi.it (C.R.); rosaria.bassi@unimi.it (R.B.); laura.cantu@unimi.it (L.C.); 2 Instituto de Investigaciones en Fisicoquímica de Córdoba (INFIQC), Consejo Nacional de Investigaciones Científicas y Técnicas (CONICET), Córdoba 5000, Argentina; 3 Department of Food and Drug, Università di Parma, Parco Area delle Scienze, 27/A, 43124 Parma, Italy; laura.bertocchi@unipr.it (L.B.); annalisa.bianchera@unipr.it (A.B.); ruggero.bettini@unipr.it (R.B.)

**Keywords:** drug delivery, light scattering, X-ray scattering, nanoparticle characterization, lung administration, mucus interaction, nanocrystals

## Abstract

The delivery of a dexamethasone formulation directly into the lung appears as an appropriate strategy to strengthen the systemic administration, reducing the dosage in the treatment of lung severe inflammations. For this purpose, a hyaluronic acid-dexamethasone formulation was developed, affording an inhalable reconstituted nanosuspension suitable to be aerosolized. The physico-chemical and biopharmaceutical properties of the formulation were tested: size, stability, loading of the spray-dried dry powder, reconstitution capability upon redispersion in aqueous media. Detailed structural insights on nanoparticles after reconstitution were obtained by light and X-ray scattering techniques. (1) The size of the nanoparticles, around 200 nm, is in the proper range for a possible engulfment by macrophages. (2) Their structure is of the core-shell type, hosting dexamethasone nanocrystals inside and carrying hyaluronic acid chains on the surface. This specific structure allows for nanosuspension stability and provides nanoparticles with muco-inert properties. (3) The nanosuspension can be efficiently aerosolized, allowing for a high drug fraction potentially reaching the deep lung. Thus, this formulation represents a promising tool for the lung administration via nebulization directly in the pipe of ventilators, to be used as such or as adjunct therapy for severe lung inflammation.

## 1. Introduction

Dexamethasone (DEX) is a synthetic glucocorticoid used in a wide range of diseases [1,2,3,4], particularly when related with lung, from occlusive airway conditions such as asthma [5] to maturation of foetus lung [6]. Recent studies report that DEX can be profitably applied to the treatment of acute respiratory distress syndrome [7] and could reduce deaths among patients with serious cases of COVID-19. Death rates drop by 1/3 among patients on ventilators and by 1/5 among patients receiving oxygen with no artificial ventilation [8]. Moreover, DEX treatment can shorten the hospitalization period ([9] and references therein). In fact, the overreaction of the immune system often occurring in patients with serious cases of COVID-19 can be suppressed by DEX, which is a strong and effective anti-inflammatory corticoid, and inflammation can be restrained.

However, despite its therapeutic efficacy, long-term systemic administration of DEX has been hampered by severe side effects such as renal failure, remarkable blood pressure reduction, weight loss, and vision impairment. In this context, there is an urgent need for new strategies to fully exploit the therapeutic potential of DEX while reducing its systemic side effects [10]. In this respect, local delivery directly to the lung in the form of liquid aerosol administered into the pipe of the ventilator [11] or with the oxygen supplement appears as a suitable strategy to parallel and strengthen the systemic administration while reducing its dosage.

The formulation of DEX in nano-carriers could improve the delivery of this insoluble drug to the targeted epithelial tissue. Different types of nanotechnologies have been developed, including the conjugation of DEX with a polymeric chain or a lipidic structure [12,13,14,15,16,17], as well as its encapsulation in solid nanoparticles, its inclusion in a solid matrix or its formulation as a small crystal evenly coated with a polymer [3,10,18]. In particular, formulations where DEX is hosted in a controlled crystalline solid core of a nanostructure show both improved solubility with respect to raw dexamethasone and an increased residence time as compared to its water-soluble analogous, dexamethasone-phosphate.

On the other hand, as a major common drawback of nanoparticle technologies, scalability is usually poor and industrial implementation difficult. Recently, Martinelli et al. [19] and Rossi et al. [20] reported on the production of nanoparticles with hyaluronic acid (HYA), in the 300–400 nm size range and efficiently co-encapsulating different active molecules, through an easily scalable antisolvent method [20]. These nanoparticles could be successfully dried to obtain a stable free-flowing powder, easy to handle and capable of regenerating nanoparticles in their original size upon contact with physiological fluids.

Notably, the efficacy of DEX may be improved by promoting its uptake through the alveolar macrophage pathway, as macrophages play an important role in the inflammatory response, provided that the nanoparticles are in the 100–500 nm size range. Encapsulation of DEX with HYA appears strategic to foster the alveolar macrophages pathway as it exploits both the suitable size of the nanoparticles [21] and the supporting nature of HYA. HYA is an anionic polysaccharide constituted of a variable number of repeating disaccharide units, namely d-glucuronic acid and N-acetyl-d-glucosamine, and most of its properties depend on its molecular size. It is present in the extracellular matrix of various tissues, such as skin, cartilage, synovia and vitreous humour, and it displays anti-inflammatory properties and plays an important role in tissue regeneration [22]. Interestingly, it is selectively captured by macrophages via the CD44 receptor, residing on the macrophage cell membrane, and if its molecular weight is relatively high, it can polarize macrophages toward an anti-inflammatory phenotype [23,24]. It has been found that while high-molecular-weight HYA displays anti-inflammatory and immunosuppressive properties, low-molecular-weight HYA is a potent pro-inflammatory molecule [25].

Thus, the combination of DEX and HYA in the form of nanoparticles for respiratory drug delivery appears a suitable approach for an adjunct therapy in lung severe inflammations, such as in the case of COVID-19, where the major efficacy of DEX is displayed in ventilated patients. The rationale of this drug delivery strategy is to increase the drug concentration in the target pulmonary tissue and in the alveolar macrophages, with concomitant reduction of the systemic exposure. Inhalation administration of drugs has already been implemented for patients receiving artificial ventilation. Aerosolized bronchodilator therapy is employed in intensive care units and some manufacturers have integrated aerosol drug delivery technology with their ventilators. In addition, several studies have been performed aiming at investigating the pharmacokinetics of antibiotics upon this type of administration [26,27].

The aim of the present work was to develop a hyaluronic acid-dexamethasone (HYA–DEX) formulation, affording an inhalable reconstituted nanosuspension, potentially suitable to be aerosolized in the gas stream of a ventilator.

A HYA–DEX nanosuspension was prepared starting from an aqueous solution of HYA, then mixed with a solution of DEX in ethanol and spray-dried to remove the solvent. A cell-free experimental approach was designed to test the HYA–DEX formulation for its physico-chemical and biopharmaceutical properties: size, stability, loading, and capability of nanosuspension reconstitution upon dispersion of the dry powder in aqueous media. We applied complementary techniques, i.e., Dynamic Laser light Scattering (DLS), Small Angle X-ray Scattering (SAXS) and Wide Angle X-ray Scattering (WAXS), to obtain detailed structural insights on reconstituted nanoparticles in aqueous solution over a wide range of length-scales, from the hundreds to the tenths of nm [28]. The fraction of HYA complexed in the reconstituted nanoparticles was quantified at different concentrations and in different solvents, namely water and phosphate buffer (PB). Finally, the structural stability of reconstituted nanoparticles against a mucus model was checked with porcine purified mucin, and the respirability of the nanosuspension was assessed using a high-performing nebulizer.

## 2. Results

**Figure 1:**
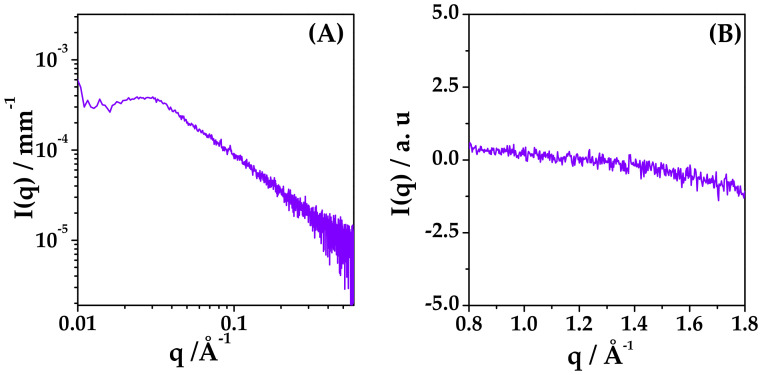
(**A**) SAXS and (**B**) WAXS spectra of ethanol/water dispersion of mixed HYA and DEX at 55: 45 mass proportion, 5 mg/mL total concentration. Ethanol/water 45:55 volume fraction.

DEX and HYA were prepared as described in Section 4.2. The ethanol/water suspension (45:55 *v*/*v* fraction) of mixed HYA:DEX at 0.06:99.94 mole fraction (55:45 mass fraction) and 5 mg/mL total concentration was observed by small and wide angle X-ray scattering (SAXS and WAXS) experiments to assess the degree of dispersion of DEX just before spray drying. Results reported in Figure 1 reveal that DEX is molecularly dispersed in the solution at this stage, as no crystalline diffraction patterns can be observed in the SAXS and WAXS spectra. In the SAXS spectrum, a single broad correlation peak is present, probably due to the spatial arrangement of HYA chains [29].

### 2.1. Powder Characterization

**Figure 2:**
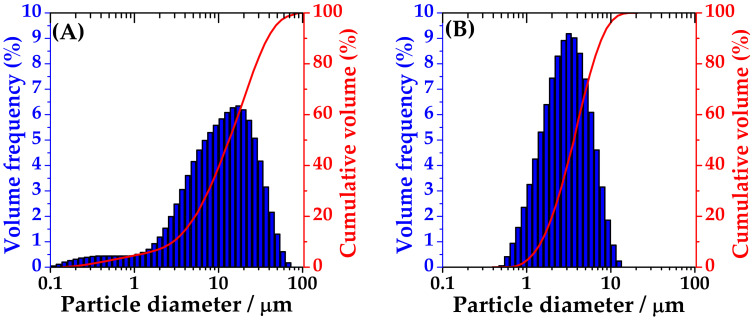
Size distribution of spray-dried particles of HYA–DEX (**A**) and only-DEX (**B**).

The particle size distribution of the spray-dried powders containing only-DEX and HYA–DEX (with sodium hyaluronate) was measured by laser diffraction. The analysis of drug-loaded-HYA microparticles reveals a relatively wide distribution with Dv(50) around 11 μm (Table 1) and (Figure 2, panel A) with a small population below 2 µm. On the contrary, microparticles obtained without the addition of the polymer presented a narrow distribution with Dv(90) well below 10 μm and Dv(50) = 2.8 (Figure 2, panel B).

**Figure 3:**
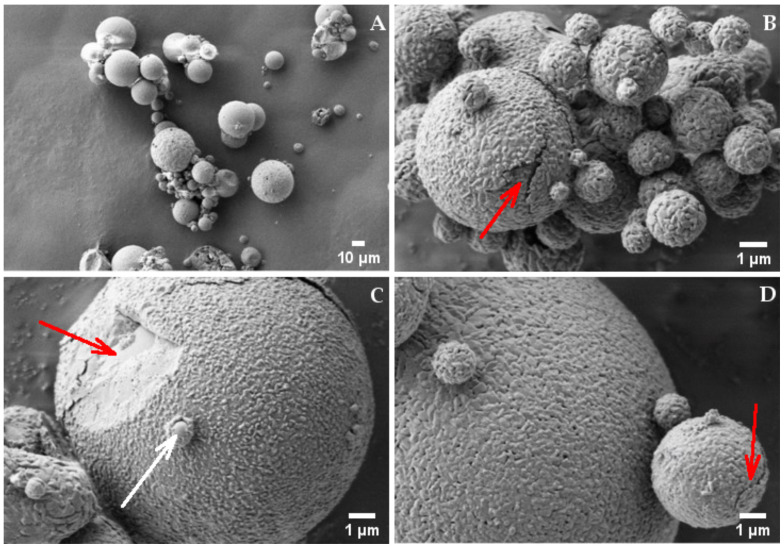
SEM picture of HYA–DEX microparticles taken at (**A**) 1000× and (**B**–**D**) 20,000× magnifications. Red arrows: (**B**,**D**) particle cracks, (**C**) wrinkled surface. White arrow: small and smooth particles, partially surrounded by the wrinkled coating.

Figure 3 reports the scanning electron microscopy pictures obtained at different magnifications from the spray-dried powder of HYA–DEX. The pictures taken at lower magnification, Figure 3 Panel A, confirm the above-mentioned size distribution; the particles present a spherical shape with an average size around 10 µm. At higher magnification, particles show a peculiar morphology, with a wrinkled surface, bread-crust looking, covering a smoother surface (red arrow in panel C). The structure of this coating is clearly visible in some particles presenting cracks (red arrows in Panel B and D), in bigger as well as in smaller particles. It is also worth noting the presence of small and smooth nanoparticles, partially surrounded by the wrinkled coating, as evidenced by the white arrow in panel C.

**Figure 4:**
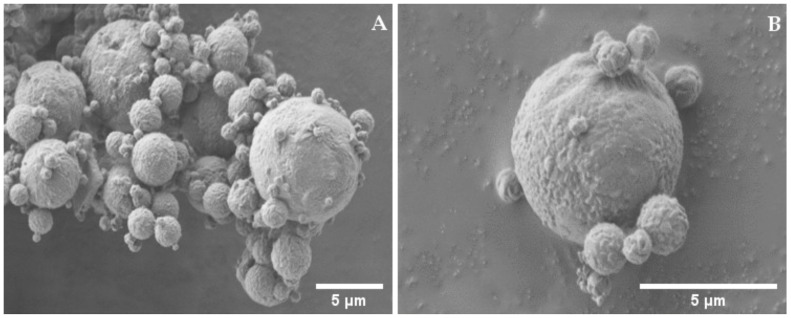
SEM picture of DEX microparticles taken at (**A**) 1000× and (**B**) 20,000× magnifications.

In addition, particles of the only-DEX powder (Figure 4) are spherical and display a wrinkled surface, but at much lower extent and without cracks (Panel B) and appear more homogeneous in size (Panel A) as compared to HYA–DEX.

Based on these observation and data reported in Figure 1, we hypothesized that the structure of the drug-polymer spray-dried particles substantially consist of a dexamethasone core surrounded by a hyaluronate shell. This hypothesis is based on considering that, during the drying process, ethanol evaporates more quickly than water, thus determining the formation of DEX nuclei, dispersed among HYA polymer chains, dexamethasone being dissolved in alcohol. The slower desiccation of HYA reasonably determines the deposition of the polymer on and among the preformed nano-sized drug cores and on the outer surface of the microparticles. HPLC quantification shows that the DEX content of the HYA–DEX powder is lower (34.3 ± 0.5% *w*/*w*) than nominal (45% *w*/*w*). These data allow for calculating a loading capacity of about 35% and a loading efficiency slightly higher than 76%. The difference between the DEX content of the HYA–DEX powder and nominal content could be explained by the loss of very small DEX particles during the drying process. In agreement with the above hypothesis, very small particles of DEX are formed before HYA can drape their surface, producing a significant increase in particle size. Before HYA deposition, the smallest DEX particles transported by the gas aspiration are not captured by the cyclone and are lost in the filter of the spray-drying apparatus.

### 2.2. Reconstituted Nanoparticles Characterization

The characterization of HYA–DEX suspension after powder redissolution was performed with laser light and X-ray scattering techniques, providing information on the size and internal arrangement of the complexes that form upon contact of microparticles with water or PB buffer.

Samples at different concentrations were prepared diluting the 1 mg/mL suspension and observed by dynamic light scattering (DLS). The mean size of particles in the reconstituted samples were calculated from the measure of the diffusion coefficient, after correction for the viscosity of the solution, higher with respect to the water one (2.5 cP at 1 mg/mL and 1.2 cP at 0.5 mg/mL), due to the presence of HYA chains [30].

In all samples, both in water and in PB, nanoparticles were formed, with a mean hydrodynamic size of 280–300 nm in water and 170–200 nm in PB buffer, as reported in Table 2, both systems being quite polydisperse, 0.3–0.4 PDI. A small fraction of bigger aggregates (few % relative volume) was always present, with the size of microns, as some microparticles did not disaggregate when redispersed.

The larger hydrodynamic size observed in salt-free water can be due to a different, more stretched, arrangement of HYA polymer chains protruding from the surface of the nanoparticles into the surrounding solvent.

Z-potential (ζ) values measured for each suspension of nanoparticles are reported in Table 2. In all samples, negative ζ values were found, in the range −20–−30 mV in PB, while below −50 mV at zero ionic strength (water).

To obtain information on the internal structure of these nanoparticles, X-ray scattering experiments were performed on redispersed HYA–DEX nanoparticles.

**Figure 5:**
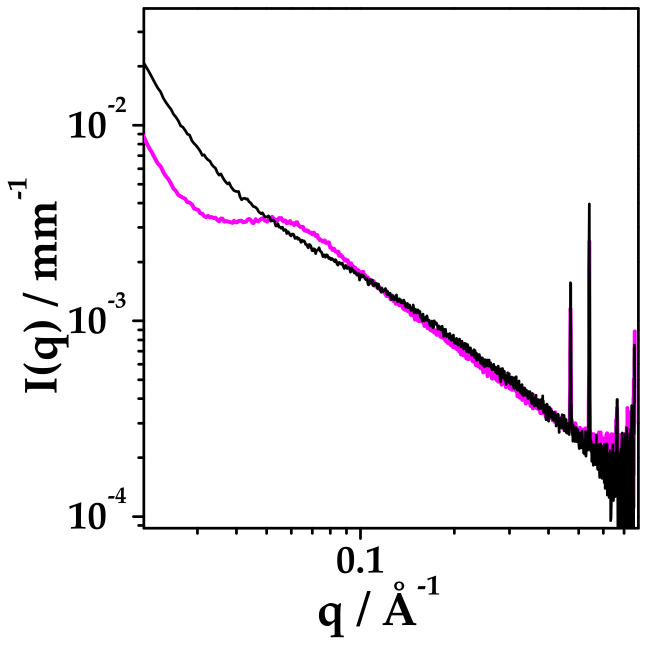
SAXS intensity profiles for HYA–DEX nanosuspension at 20 mg/mL dispersed in water (magenta) or phosphate buffer (black).

Preliminary SAXS experiments on HYA–DEX nanoparticles reconstituted in water and PB at 20 mg/mL (see Figure 5) revealed that the scattered intensity profiles account for two contributions, one due to nanoparticles, the other to the fraction of HYA that is not complexed in the nanoparticles but free in solution. In particular, a characteristic peak is visible in the spectrum of the salt-free sample at q = 0.058 Å^−1^ (see Figure 5), that is not displayed by the buffered suspension. This peak, already observed in the ethanol/water suspension before spray-drying, arises from HYA organization in water. In fact, in polyelectrolyte solutions, including polysaccharide chains, this peak was seen to be mainly modulated by repulsive electrostatic interactions, screened in presence of salt [29]. The correlation peak reflects the pseudo-periodic organization of polyelectrolyte chains in solution, resulting from inter-chain repulsive interactions.

To evaluate the fraction of the free HYA in both environmental conditions and to enucleate the structural features of the nanoparticles, we performed SAXS experiments on the nanoparticles reconstituted at different concentrations in salt-free and in PB buffer (138 mM salt concentration) and on HYA solutions in the appropriate range of concentration, corresponding to the amount of HYA in the different reconstituted samples.

### 2.3. Characterization of Hyaluronic Acid in Water and PB Solution

**Figure 6:**
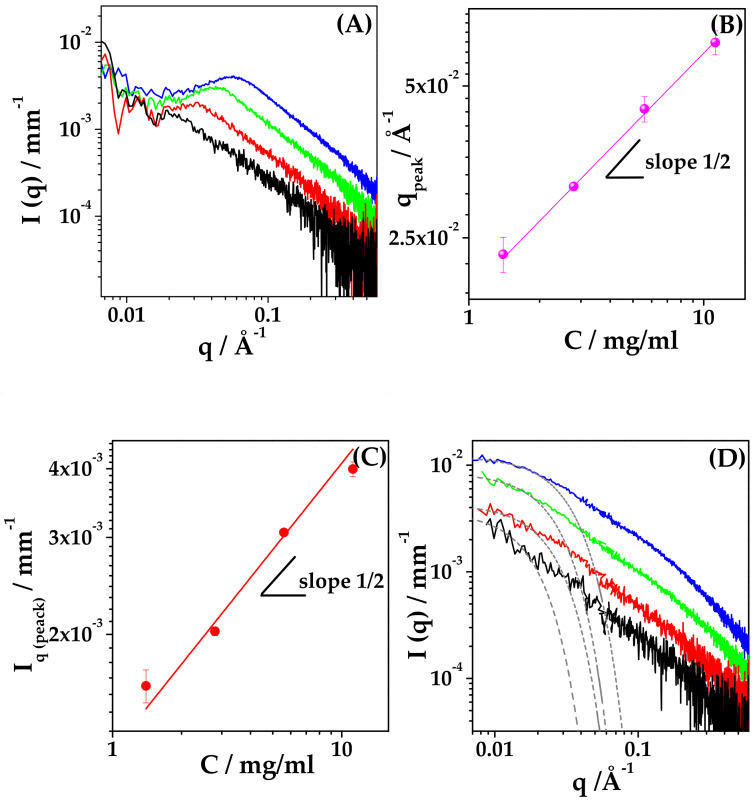
SAXS of HYA solution. HYA concentration: 1.4 mg/mL (black), 2.8 mg/mL (red), 5.6 mg/mL (green) and 11.2 mg/mL (blue). Water: (**A**) Intensity profiles at increasing concentration; (**B**) Scaling behaviour of the correlation peak position (q_peak_); (**C**) Scaling behaviour of the scattered intensity at q = q_peak_, I (q_peak_). PB: (**D**) Intensity profiles for HYA in PB (138 mM salt concentration) and the corresponding Guinier Fit (gray dash line).

We prepared water solutions of HYA in the range of concentration of interest, varying between 1.4 and 11.2 mg/mL. The measured SAXS intensity profiles are reported in Figure 6 (Panel A), showing the characteristic intensity peak for polyelectrolytes in salt-free solution [31]. The q value of the correlation peak (q_peak_) is related to the inter-chain characteristic distance ξ according to q_peak_ = 2π/ξ. On increasing the concentration of HYA, the measured correlation peak shifts to higher q values, corresponding to shorter inter-chain characteristic distances, as reported in Table 3 (ξ values). The values of q_peak_ are reported as a function of HYA concentration c in Figure 6 (panel B) to assess the scaling behaviour of the polymer, in the investigated range of concentration. The value of q_peak_ scales with c^1/2^, revealing that in salt-free solution HYA behaves as a classical hydrophilic polyelectrolyte in all the explored concentration interval. The long-range electrostatic interactions govern the conformation of HYA, which arranges in elongated rod-like chains, as reported for HYA with similar molar mass [22].

The scaling behaviour of the intensity of the scattered radiation at q = q_peak_, I(q_peak_), is also shown in Figure 6 (panel C). The variation of the peak intensity is proportional to c^1/2^, as predicted by the model of Koyama [32] for wormlike chain polymers with strong electrostatic interactions.

Finally, the trend of I(q) = q^−s^ in the high-q range (q > 0.2–0.3 Å^−1^) was determined from the spectra of HYA in water, and the values of the exponents are reported in Table 3, showing a similar value around 1.3, as expected for a rod-like conformation of HYA in salt-free solution, as already reported in literature [22,33].

Parallel SAXS experiments on solutions of HYA in PB were performed, and the measured spectra are reported in Figure 6 (panel D). The intensity profiles differ from the corresponding ones obtained from salt-free samples, in both the low-q and high-q regions, and show the disappearance of the polyelectrolyte correlation peak.

When dissolved in PB solutions, the conformation of HYA chains is no longer solely governed by electrostatics, and the polymer can be modelled as a beaded necklace [22] with globular denser regions. The mean gyration radius, R_g_, of denser regions was calculated using a Guinier law I(q) = I_0_ exp(−q^2^R_g_^2^/3), and is listed in Table 3. The size depends on HYA concentration: R_g_ increases from 54 to 98 Å upon dilution, as expected for a vanishing effect of electrostatics in the case of diluted solutions of polyelectrolytes in the presence of non-zero ionic strength.

The trend of I(q) = q^−s^ at high-q values was determined from the spectra of HYA in PB, and the corresponding values of the exponents are reported in the last column of Table 3, showing that s is around 1.3, similar to the salt-free condition, at c < 3 mg/mL, while it increases to 1.5 at c > 3 mg/mL. Then, HYA slightly deviates from the rod-like arrangement and its polymer chain evolves to a more compact conformation. Still, its conformation remains quite rigid and the polymer does not assume a random walk arrangement, characterized by I(q) = q^−2^ (s = 2) [34].

### 2.4. Evaluation of the Fraction of Bound/Unbound HYA in the Reconstituted Nanoparticle Suspension

Results on HYA conformation in solution allowed for the quantification of the free and complexed HYA fractions in nanoparticles solutions after reconstitution of dry microparticles.

**Figure 7:**
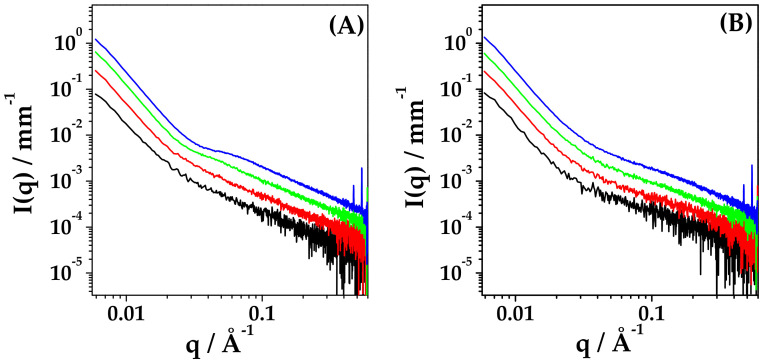
SAXS spectra of HYA–DEX nanoparticles 0.55:0.45 weight fraction at different concentrations in salt-free water (**A**) and in PB (**B**). HYA–DEX concentration: 2.5 mg/mL (black), 5 mg/mL (red), 10 mg/mL (green) and 20 mg/mL (blue).

Figure 7 reports the intensity profiles of HYA–DEX nanoparticles (0.55:0.45 weight fraction) at different concentrations in salt-free water and in PB (panel A and panel B, respectively). In panel A, the characteristic correlation peak of free HYA in salt-free solution is visible at 10 and 20 mg/mL, shifting at larger q-values upon concentration increase. In Supplementary Material (Figure S1), the comparison between the spectra of HYA–DEX nanoparticles with those of HYA at the same concentration as in the nanoparticles solution is reported for all investigated systems, showing differences both in the peak position and in the intensity at high-q values. These differences suggest that a fraction of the admixed HYA is involved in the formation of HYA–DEX nanoparticles.

The exact evaluation of the fraction of free and complexed HYA is not trivial and was performed with parallel procedures to reach a reliable result.

**Figure 8:**
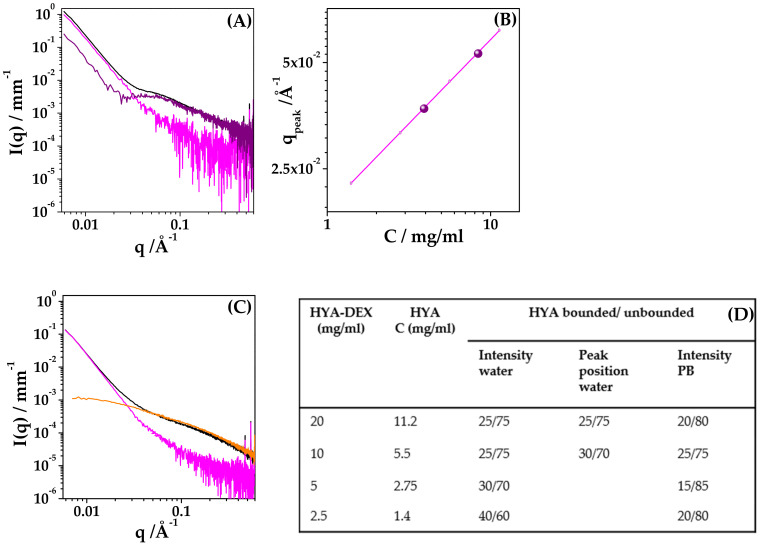
Evaluation of bound/unbound HYA. (**A**) SAXS spectra of HYA–DEX nanoparticles at 20 mg/mL in water (black), experimental intensity contribution of nanoparticles (magenta), difference between black and magenta signals (purple). (**B**) Peak position (q_peak_) for 10 mg/mL and 20 mg/mL samples (purple dots). The magenta line represents the scaling behaviour of HYA. (**C**) SAXS spectra of HYA–DEX nanoparticles at 20 mg/mL in PB (black), intensity contribution of HYA at the same nominal concentration, 11.2 mg/mL (orange), difference between black and 0.8× orange signals (magenta) to obtain the contribution of nanoparticles in PB. (**D**) (Table) Fraction of bound/unbound HYA as calculated by the analysis of the intensity contribution or by the q_peak_ position.

(a) At c = 2.5 and 5 mg/mL, the deviation of the amount of free HYA from the nominal concentration is mainly visible as a difference in the intensity contribution at q > 0.06 Å^−1^. A fraction of the free-HYA spectra was subtracted from the HYA–DEX ones to obtain similar intensity profiles, rigidly scaled in absolute intensity, as for identical nanoparticles at two different concentrations (Figure S2). The fraction of bound/unbound HYA, reported in the table of Figure 8, is 30/70 and 40/60 for the samples at 5 mg/mL and 2.5 mg/mL, respectively. The experimental intensity profiles of HYA–DEX nanoparticles, obtained as described above, were then subtracted, after normalization for concentration, from the spectra of the 10 mg/mL and 20 mg/mL solutions, to obtain the intensity contribution of the fraction of unbound HYA, as reported in Figure 8A for the 20 mg/mL sample (purple signal in the graph).

(b) For the samples at higher concentration, the correlation peak is visible, and its position can be determined and compared to the scaling law found for HYA in salt-free solution, Figure 8B. The experimental data (dots) lie, on the scaling curve of HYA, at concentrations lower than the nominal ones. The actual concentration values of bound/unbound HYA are 25/75 for both the 10 mg/mL and 20 mg/mL solutions, as reported in Figure 8 (Table D).

(c) Finally, the absolute intensity of the contribution of unbound HYA in the high-q region also was checked for samples at 10 mg/mL and 20 mg/mL, to validate the quantification procedure. Results, reported in the Table D of Figure 8, are in good agreement with the values obtained by the peak position evaluation.

For the HYA–DEX solutions in PB that do not show the correlation peaks, the fraction of bound/unbound HYA was evaluated by the intensity discrepancy in the q > 0.06 Å^−1^ region, Figure S3. The contribution of complexed-HYA in the solutions of HYA–DEX nanoparticles is about 20%, lower than the nominal content of HYA, as estimated by subtraction to obtain intensity profiles realistic for nanoparticles.

The fraction of HYA directly involved in the HYA–DEX nanoparticle reconstituted in PB is slightly lower than in salt-free water, suggesting an influence of the environmental conditions on the final amount of HYA associated with DEX upon redispersion of microparticles. The presence of salt in the solvent can disturb the hydrogen bonding formation (DEX/HYA or HYA/HYA), allowing higher desorption of HYA from the surface of the nanoparticle when dispersed in PB.

### 2.5. Internal Structure of Reconstituted Nanoparticles

**Figure 9:**
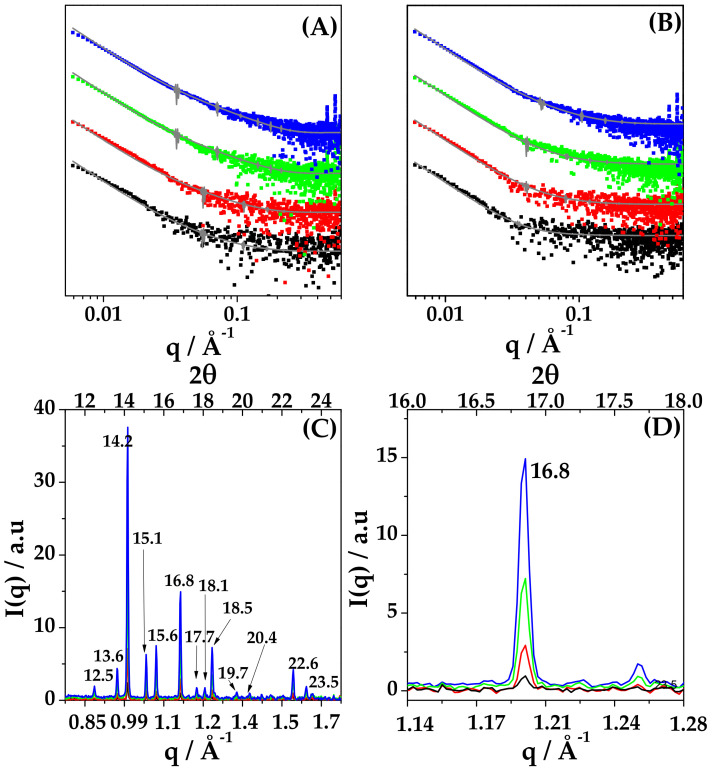
(**A**,**B**). SAXS intensity profiles of the HYA–DEX nanoparticles after subtraction of the intensity contribution of unbound HYA in water (**A**) and in PB (**B**). Lines are the fit with a core-shell polydisperse spherical model. Note: the intensity profiles were re-scaled for a better visualization. (**C**,**D**) WAXS spectra of HYA–DEX nanoparticles in water in the full q range (**C**) and in the region of one diffraction peak (**D**) to better visualize the increase of the intensity with concentration. Labels report the position of peaks expressed in 2θ. HYA–DEX concentration: 1.4 mg/mL (black), 2.8 mg/mL (red), 5.6 mg/mL (green) and 11.2 mg/mL (blue).

Figure 9 reports the SAXS intensity profiles of the HYA–DEX nanoparticles after subtraction of the intensity contribution of unbound HYA. The intensity decays are similar both at different concentrations and in different redispersion solvent. The spectra have been modelled to a polydisperse core-shell sphere and the fitting curves are reported in Figure 9A. A core of about 200 nm (scattering length density 11.8 10^−6^Å^−2^) is surrounded by a thin shell, about 1.5 nm in thickness, characterized by a high scattering length density (16 10^−6^Å^−2^), as for polysaccharide chains. Details on the parameters are reported in Supplementary Material Table S1.

SAXS results indicate that, on the mesoscale, the structure of the nanoparticles is characterized by a DEX-rich core stabilized in suspension by the presence of HYA chains, which are partially embedded in the core while partially surrounding the surface.

This hypothesis is corroborated by features on the local scale, as assessed by parallel SAXS and WAXS measurements. In all the SAXS spectra, a sequence of narrow peaks is present in the high-q region (q_1_ = 0.47 Å, q_2_ = 0.54 Å), as can be easily visualized in Figure 7, Panels A and B, for the 20 mg/mL sample. Moreover, the intensity scattered at wide angles (WAXS) was measured on the same samples and the spectra are reported in Figure 9 Panel C, showing characteristic diffraction patterns. The position of peaks is identical for all the investigated systems, while the intensity of each peak scales with concentration, as shown in Figure 9D. The experimental pattern was compared with the powder diffraction pattern of DEX reported in literature [35]. The experimental crystal structure is identical to the one reported for DEX, with an orthorhombic geometry, with lattice parameters a = 10.36 Å, b = 16.16 Å, c = 23.20 Å [36]. This local internal arrangement is also preserved when nanoparticles are reconstituted in PB buffer, as shown in Supplementary Material (Figure S4).

Results indicate that the internal structure of nanoparticles is constituted by DEX nanocrystals complexed into the globular core.

### 2.6. Stability of HYA–DEX Nanoparticles in Mucus Model

To assess the stability of nanoparticles in mucus model, SAXS experiments were performed on systems after the addition of mucin at 0.5% and 1% *w/v* final concentration. Spectra of mucin in PB at different concentrations are reported in the Supplementary Material (Figure S5), showing the characteristic features of mucin in this range of concentration, when homogeneously dispersed [37].

**Figure 10:**
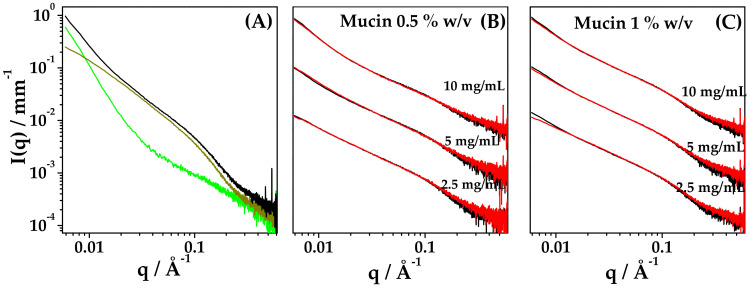
Stability of nanoparticles in mucus model. SAXS intensity profiles. (**A**) 10 mg/mL HYA–DEX nanodispersion (green), 1% *w/v* mucin (dark yellow) and admixed 10 mg/mL HYA–DEX nanoparticles dispersed in 1% *w/v* mucin (dark yellow). (**B**,**C**) Experimental profiles (black) and reconstructed profiles (red) of HYA–DEX nanoparticles dispersed in mucin; mucin concentration (**B**) 0.5% *w/v* and (**C**) 1% *w/v*. HYA–DEX concentration: 2.5 mg/mL; 5 mg/mL and 10 mg/mL. In panel (**B**,**C**), the intensity profiles were rescaled to a better visualization.

Figure 10, panel A, shows the intensity profile of admixed Mucin 1% *w/v* + HYA–DEX nanoparticles at 10 mg/mL final concentration, compared with the spectra of the two components at the same concentration in PB. Most of the graph of the intensity signal is dominated by the contribution of mucin, except for the low-q and high-q regions of the spectrum. The spectra of admixed samples, at different concentrations of nanoparticles (10, 5, 2.5 mg/mL) both in 0.5% *w/v* and 1% *w/v* mucin, were analyzed by comparing them with the scattering signal obtained by the linear combination of the intensity profiles of the single components, i.e., nanoparticles and mucin, as reported in panels B and C of Figure 10. At 0.5% *w/v* mucin, such procedure allows reconstructing the admixed spectra over the whole q-range, as for a mixture of non-interacting components. At 1% *w/v* mucin, a slight deviation of the linear-combination reconstruction from the experimental signals is visible in the low-q region only at 2.5 mg/mL concentration of nanoparticles.

Results indicate that nanoparticles are stable and do not change their structure when in interaction with mucin. The presence of HYA chains on the external shell of nanoparticles and the presence of unbound HYA in solution (both water and buffer) could be responsible for the muco-inert properties of the nanoparticles, preventing the adhesion of mucin chains to the surface.

### 2.7. In Vitro Aerosolization

**Figure 11:**
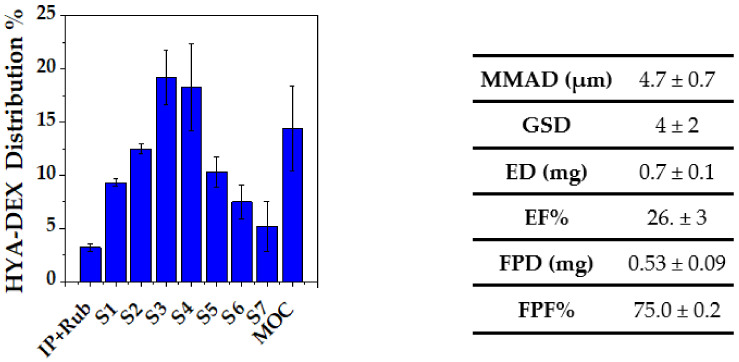
HYA–DEX distribution in the NGI apparatus upon nebulization. The table reports the aerodynamic parameters calculated from the distribution values: MMAD = Median Mass Aerodynamic Diameter; GDS = Geometric Standard Deviation; ED = Emitted Dose; EF = Emitted Fraction; FPD = Fine Particle Dose; FPF = Fine Particle Fraction.

Figure 11 reports the HYA–DEX distribution within the NGI apparatus, along with the relevant aerodynamic parameters. The emitted fraction was around 25% of the loaded dose, in agreement with the typical performance of this type of nebulizer [38]; on the other hand, the respirability of the emitted dose was significant (FPF = 75%). This is justified by a median aerodynamic diameter < 5 µm and indicates good de-aggregation properties of the nanosuspension reconstituted from the spray-dried powder. The relatively low emitted fraction may represent a limit that however, in the proposed final application, could be overcome by nebulizing directly the nanosuspension in the pipe of a ventilator.

## 3. Conclusions

A suspension consisting of complexed nanoparticles made of Hyaluronic acid and Dexamethasone can be easily obtained by reconstitution in an aqueous medium, starting from a spray-dried powder, with a scalable protocol. The reconstituted nanoparticles display both the suitable size (200–250 nm) and the putative synergic composition to foster the alveolar macrophages pathway in the reduction of lung inflammation.

This HYA–DEX nanosuspension can be aerosolized by means of a high-efficiency nebulizer, giving rise to a high drug fraction potentially reaching the deep lung. This positive aerosolization performance is the result of the peculiar structure of the suspended nanoparticles, which turned out to be constituted by a core of DEX nanocrystals stabilized by a network of HYA, mainly located at the interface with the solvent and making the first-stage biological identity of the nanoparticle. This specific feature both confers great physical stability to the nanosuspension and likely may improve the interaction of the drug with the target. In addition, it allows stability against mucus without mucoadhesion, which is a further positive aspect for the proposed application.

In conclusion, we can state that the formulation developed and investigated in the present work represents a very promising tool for the lung administration via nebulization directly in the pipe of a ventilator, bypassing the problematic systemic route, or complementing the parenteral administration as an adjunct therapy in patients in critical conditions, with severe lung inflammation.

## 4. Materials and Methods

### 4.1. Materials

Commercial mucin from porcine stomach (Type III, bound sialic acid 0.5–1%), potassium dihydrogen phosphate (KH_2_PO_4_) and sodium hydroxide (NaOH) were purchased from Sigma-Aldrich^®^ (STL, MO, USA). Dexamethasone was purchased by Metapharmaceutical (Barcelona, Spain) and sodium hyaluronate with molecular weight 750–1000 kDa by Contipro (Praha, Czech Republic). Ultrapure water was obtained by inverse osmosis using a Arium Comfort^®^ system by Sartorius (Gottingen, Germany). Ethanol, cyclohexane, and acetonitrile were of analytical grade.

Phosphate buffer pH 7.4 (PB) was prepared as indicated in the pharmacopeia [39] from KH_2_PO_4_ (200 mM) and NaOH (100 mM) solution; the final concentration was 138 mM. The solution pH was adjusted to 7.4 with NaOH.

Nanoparticle powder resuspension: The corresponding mass of nanoparticle powder was redissolved on water or PB, after sonication for 15 min to obtain a homogeneous dispersion. The concentration range of dispersions analyzed was 0.125 mg/mL–1 mg/mL for DLS and 2.5 mg/mL–20 mg/mL for SAXS and WAXS experiments.

Hyaluronic acid solutions: The corresponding mass of hyaluronic acid was dissolved in water or PB, and the measurements were performed after 30 min to allow the complete dissolution of the polymer. The range of concentration analyzed was 1.4 mg/mL–11.2 mg/mL.

All the solvents were filtered through a polycarbonate membrane with a 0.2 µm pore diameter (Whatman ^®^ Nuclepore^TM^, Madiston, UK).

### 4.2. Methods

#### 4.2.1. Production of Dexamethasone-Loaded Sodium Hyaluronate Nanoparticles

Dexamethasone nanoparticles were obtained by anti-solvent precipitation using water as anti-solvent. Water was added dropwise to an alcoholic solution of drug at 5 mg/mL. Then, 180 mg of dexamethasone was first dissolved in 36 mL of ethanol, and 29.5 mL of water was added dropwise to the alcoholic solution to promote drug precipitation. The anti-solvent was added manually, dripping 7 mL of water at a time and leaving 3 min of rest between each addition. During the precipitation, the temperature was maintained at 25 °C using a thermostatic bath and the suspension was kept under stirring at 165 rpm with a magnetic stirrer. Separately, 220 mg of sodium hyaluronate 750–1000 kDa was dissolved in 14.5 mL of ultrapure water and then added in one shot to the dexamethasone nanosuspension, reaching the final anti-solvent concentration of 55% (*v/v*). The nanosuspension was left at room temperature under stirring at 280 rpm for 30 min before spray drying.

To produce dexamethasone nanoparticles without the addition of sodium hyaluronate, the same procedure was followed, but 14.5 mL of ultrapure water was added in one shot to the drug nanosuspension without previously solubilizing the polymer.

#### 4.2.2. Spray Drying

Nanosuspensions containing dexamethasone and dexamethasone/sodium hyaluronate were dried using a Mini Spray Dryer B-290 (Büchi, Switzerland) connected with an Inert Loop B-295 for organic solvents. The needle was 0.7 mm in diameter. Inlet temperature was fixed at 100 °C and recorded outlet temperature was 68 °C. The aspirator was set at 35 m^3^/h, the feed rate of the nanosuspension at 2 mL/min; nitrogen was used as nebulizing gas, at 473 L/h.

#### 4.2.3. Aerosolization In Vitro

For the tests of aerosolization in vitro, 10 mg of powder was resuspended in 4 mL of ultrapure water to obtain a final concentration of 2.5 mg/mL. The suspension was sonicated for 15 min and loaded into the ampoule. As a nebulizing device, a PARI LC-Sprint ampoule and the compressor PARI Turbo Boy (Pari, Starnberg, Germany) were used. The air flow was fixed at 15 L/min and the compressor was activated 30 s after turning on the aspiration. The nebulization was carried out until sputtering (about 16 min) and at the end of the aerosolization the aspiration was stopped 5 s after the shut-down of the compressor. The nebulizer was connected through a rubber adaptor to a Next Generation Impactor (Copley Scientific, UK) composed by seven stages. After nebulization the ampoule, the induction port (IP), the rubber adaptor, the 7 stages and the micro-orifice collector (MOC) were washed with 5 mL of ethanol-water (30:70 *v/v*) and the collected solutions were sonicated for 15 min. Before HPLC analysis, MOC-solution was filtered with a cellulose acetate filter with a cut-off of 0.45 μm. The mass median aerodynamic diameter (MMAD) and Geometric standard deviation (GSD values were obtained by plotting in ordinate on probit scale the cumulative undersize percentage of drug collected and in abscissa the logarithm of cut-off values for each stage at 15 L/min. MMAD is the value corresponding to the 50% in the plot; GSD is the value corresponding to the square root of the ratio between the diameters when the cumulative mass is equal to 85% and 16%. The emitted dose (ED) was obtained by the sum of dexamethasone collected from the IP to the MOC of the NGI, while the emitted fraction (EF%) was calculated as percentage of the ED on the total amount of drug recovered at the end of the aerosolization. The amount of drug with an aerodynamic diameter below 5 μm, the fine particle dose (FPD), was obtained by the sum of drug collected from stage 3 to the MOC of the NGI, while the respective fine particle fraction (FPF%) was calculated as the percentage of the FPD on the ED.

#### 4.2.4. High Pressure Liquid Chromatography (HPLC) for Determination of Dexamethasone in Microparticles

A C18 column (150 × 4.6 mm, 5 μm) was employed for the quantification of dexamethasone by HPLC, using an Agilent 1200 series LC (Agilent, Santa Clara, CA, USA) at a wavelength of 254 nm. The mobile phase was a mixture of acetonitrile (ACN) and water pumped at 1.2 mL/min according to the following gradient: 0–2.7 min ACN 40%; 2.7–10 min ACN 100%; 10 min–12 min ACN 40%. The column was maintained at 45 °C and sample injection volume was 20 μL. The run time was of 12 min and DEX retention time was 2.5 min.

#### 4.2.5. Powders Characterization

Laser diffraction was employed to investigate the particle size distribution of the dried powders. Powder samples were dispersed in cyclohexane at a concentration of about 1 mg/mL. The suspensions were analyzed after the addition of 0.1% Span^®^ 85 and 5 min of sonication using a Spraytec^®^ granulometer (Malvern, UK).

The morphology of the powders was evaluated by scanning electron microscopy using a FESEM SUPRA™ 40 (Carl Zeiss, Jena, Germany). Each powder sample was deposited on adhesive black carbon tabs pre-mounted on aluminum stubs to allow the dispersion of the charge and coated with a gold film of about 60 nm. The particles in excess were gently removed with a nitrogen flow. The samples were analyzed under high vacuum conditions (1.33 × 10^−2^ Pa for 30 min) and the images collected at different magnifications (1 K X, 10 K X and 20 K X) using an accelerating voltage of 1 kV.

#### 4.2.6. Laser Light Scattering

The measure of the particle molecular mass and size distribution was carried out using light-scattering techniques. Static and dynamic laser light scattering experiments were performed on a home-made apparatus, equipped with a Nd-Yag laser source (532 nm) and four independent photomultipliers at 90°. All samples were submitted to parallel and independent Static and Dynamic Laser Light Scattering (SLS and DLS) measurements at 25 °C. The mean scattered intensity was acquired for the nanoparticles solutions and for HYA solutions in a dilute regime along a dilution line (1:2 1:4 1:8 with respect to the original formulations 0.1% *w/v*) to check for the presence of inter-particle interaction. Parallel acquisition of the intensity correlation function by Dynamic Laser Light Scattering (DLS) gave the mean translational diffusion coefficient of the particles and then, via the Stokes–Einstein equation, their hydrodynamic diameter. DLS data analysis was carried out using the non-negative least squares (NNLS) method [40], suitable to determine the size distribution of the particles.

#### 4.2.7. Zeta Potential

The evaluation of the Z-potential (ζ) of nanoparticles in suspension was performed measuring the electrophoretic mobility with a ZetaPlus and ZetaPals analyzer from Brookhaven Instruments (Holtsville, NY, USA). The analyses were performed both in salt-free and PB buffer suspensions at 25 °C. Five measurements were repeated and averaged on each sample to obtain the mean ζ value.

#### 4.2.8. X-ray Scattering

SAXS and WAXS: experiments were performed on the ID02 beamline at ESRF (Grenoble, France) and on the SAXS beamline at ELETTRA (Trieste, Italy). The configuration with a flow-through cell was selected, which allowed the measurements of the samples and of the reference in identical conditions. The scattered intensity was acquired in the q range 7.10^−2^ ≤ q ≤ 0.7 Å^−1^ for SAXS and 1 ≤ q ≤ 2 Å^−1^ for WAXS.

Ten short frames (0.1 s ESRF, 1 s ELETTRA) were acquired and averaged, after check, to avoid any radiation damage. After data normalization and correction, the cell and solvent contributions were subtracted from each spectrum to obtain the excess scattered intensity in absolute units I(q), mm^−1^.

Data analysis was performed using SasView 4.2.1 software [41].

## Figures and Tables

**Table 1:** Volume-weighted diameters (μm) at 10th, 50th and 90th percentiles obtained from the cumulative curve of spray-dried powder of DEX alone or with HYA.

Size (μm)	HYA–DEX	Only-DEX
D_v_(10)	2.5 ± 0.1	1.19 ± 0.06
D_v_(50)	11.2 ± 0.2	2.8 ± 0.2
D_v_(90)	31.7 ± 0.6	6.3 ± 0.2

**Table 2:** Hydrodynamic diameter (D_H_) measured by DLS and Z- potential (**ζ**) at different concentrations of HYA–DEX nanoparticles resuspended in water or phosphate buffer (PB). Polydispersity index is around 0.3–0.4. Results in water are viscosity corrected, * 1.2 cps, ** 2.5 cps.

HYA–DEX Nanoparticles mg/mL	D_H_ in Water nm	ζ in Water mV	D_H_ in PB nm	ζ in PB mV
0.125	280	−61 ± 4	210	−28 ± 5
0.25	290	−60 ± 3	170	−22 ± 6
0.5	280 *	−57 ± 3	170	−27 ± 7
1	300 **	−57 ± 1	200	−23 ± 6

**Table 3:** HYA in solution. Characteristic exponents in water and PB, inter-chain characteristic distance ξ, mean gyration radius R_g_.

Concentration mg/mL	ξ/Å	s Water	R_g_ PB /Å	s PB
1.4	270	1.3	98	1.3
2.8	220	1.3	70	1.3
5.6	140	1.3	68	1.5
11.2	110	1.3	54	1.5

## Data Availability

Data is contained within the article or Supplementary Materials.

## Supplementary Materials

The following are available online at https://www.mdpi.com/article/10.3390/ijms221910480/s1.
