# Supplementary material for: Hyaluronic Acid—Dexamethasone Nanoparticles for Local Adjunct Therapy of Lung Inflammation
Source: Int J Mol Sci. 2021 Sep 28;22(19):10480. doi: 10.3390/ijms221910480 (PMC8509068; doi:10.3390/ijms221910480)
Supplement: Supplementary file 1 [file ijms-22-10480-s001.zip › ijms-1373294-supplementary.pdf]

Supplementary Materials

Hyaluronic acid - dexamethasone nanoparticles for local adjunct therapy of lung inflammation

Candelaria Ines Camara[1,3#], Laura Bertocchi[2#], Caterina Ricci[1], Rosaria Bassi[1], Annalisa Bianchera[2], Laura Cantu'[1], Ruggero Bettini[2], Elena Del Favero[1*]

[1] BIOMETRA Dept., Università degli studi di Milano, LITA, V.le F.lli Cervi 93, 20090, Segrate, Italy.
[2] Dept. of Food and Drug, Università di Parma, Parco Area delle Scienze, 27/A - 43124 Parma, Italy.
[3] Consejo Nacional de Investigaciones Científicas y Técnicas (CONICET), Instituto de Investigaciones en Fisicoquímica de Córdoba (INFIQC), X5000HUA, Córdoba, Argentina; candelaria_camara@unc.edu.ar.
* Correspondence: Elena Del Favero, elena.delfavero@unimi.it, BIOMETRA Dept., Università degli studi di Milano, LITA, V.le F.lli Cervi 93, 20090, Segrate, Italy.
[#] These authors contribute equally to the work.

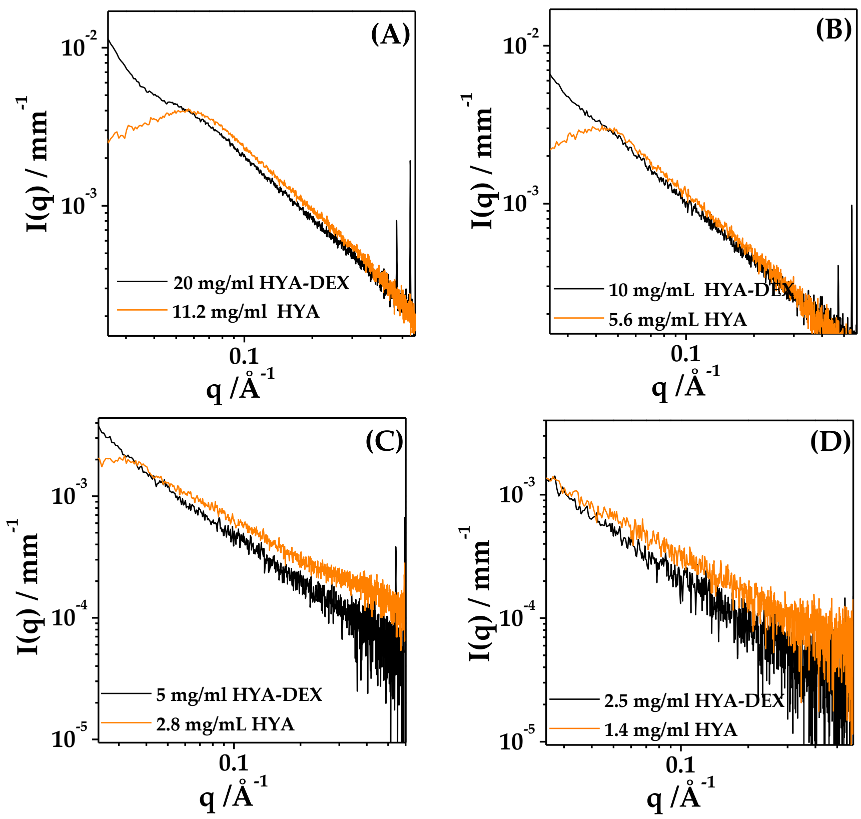

Figure S1. Comparison between the SAXS spectra of HYA-DEX nanoparticles (black lines) with the ones of HYA (orange lines) at the same concentration present in each solution of nanoparticles for all investigated systems, showing differences both in the peak position and in the intensity at high q values.

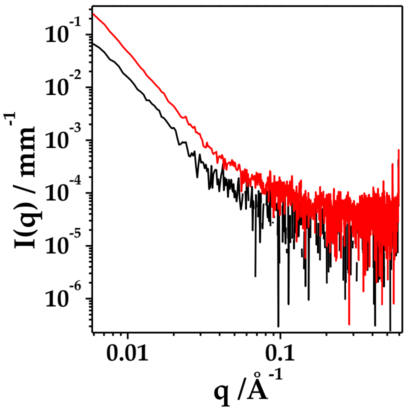

Figure S2. SAXS spectra of HYA-DEX nanoparticles obtained after subtraction of the scattered intensity contribution of unbounded HYA.

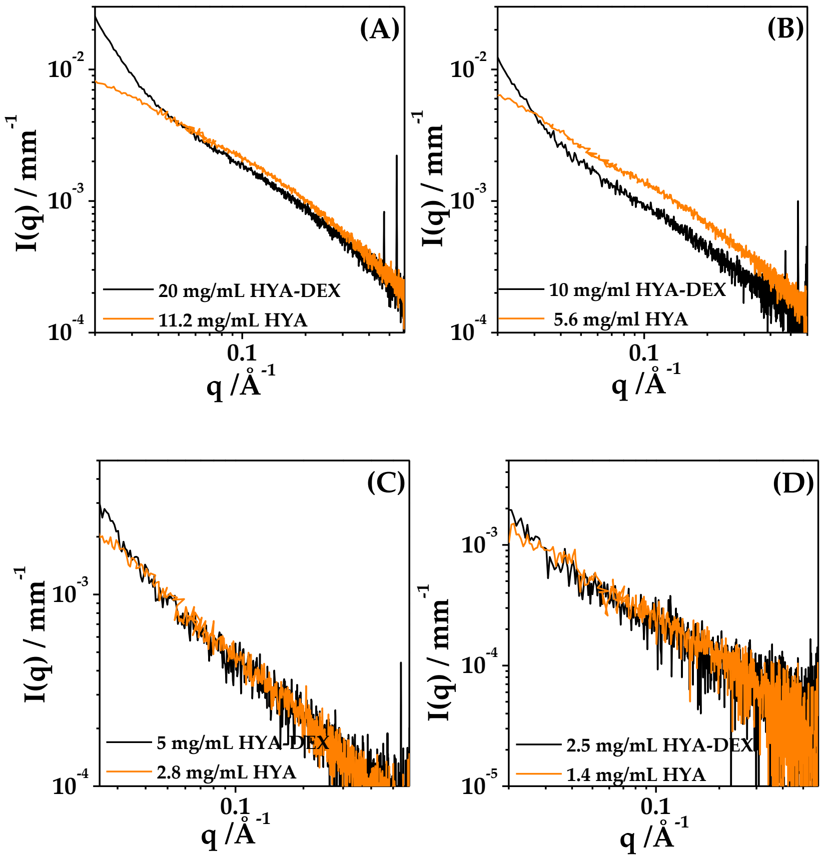

Figure S3. Comparison between the SAXS spectra of HYA-DEX nanoparticles (black lines) with the ones of HYA (orange lines) at the same concentration present in each solution of nanoparticles for all investigated systems in PB, showing differences in the intensity at high q values.

Core-shell model and fitting parameters

The shape of the scattering curves was modeled using the form factor P(q) of polydisperse core-shell spherical particles (i = 2).

$$P(q) \div \left[\sum_{i=1}^{4} 3V_i \, (\rho_i - \rho_{i+1}) \frac{\sin(qR_i) - qR_i \cos(qR_i)}{(qR_i)^3} \right]^2$$

where V_i and R_i indicate the volume and radius of the concentric spheres, and ρ_i are the scattering length densities (SLDs) of the core (i = 1) and of the shell (i = 2); ρ_3 is the scattering length density of the solvent. The polydispersity of the particle size was determined based on Schultz distribution, 0.2 for all systems.

Table S1: Parameters corresponding to the fit shown in Figure 9 A-B, with core-shell polydisperse spherical model. The polydispersity was fixed at 0.2. Scattering length density values used for fitting.

	**C mg/ml**	**Core Radius nm**	**Shell Thickness nm**
Salt-free Water	2.5	160	1.5
	5	141	1.3
	10	250	1.5
	20	250	1.5
Phosphate Buffer (PB)	2.5	250	1.0
	5	224	1.2
	10	220	1.1
	20	170	1.2

	**SLD Å^{-2}**
Water	9.46 10^{-6}
Dexamethasone	11.8 10^{-6}
Hyaluronic acid	16 10^{-6}

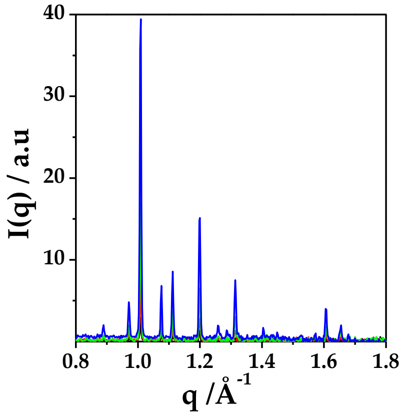

Figure S4. WAXS spectra of HYA-DEX nanoparticles in phosphate buffer.

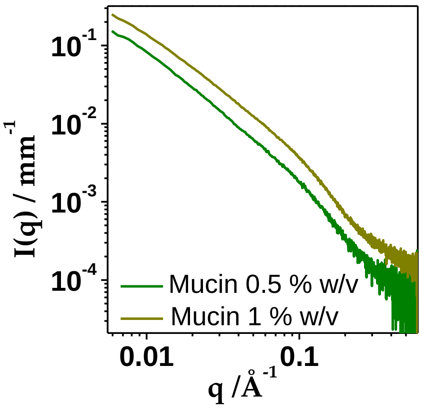

Figure S5. SAXS spectra of mucin at concentration 0.5% w/v and 1% w/v in phosphate buffer.
